# CNOT4 enhances the efficacy of anti‐PD‐1 immunotherapy in a model of non‐small cell lung cancer

**DOI:** 10.1002/2211-5463.12998

**Published:** 2020-11-05

**Authors:** Biao Zhang, Song Han, Haitao Ma, Shaomu Chen

**Affiliations:** ^1^ Institute of Thoracic Surgery the First Affiliated Hospital of Soochow University Suzhou China; ^2^ Department of Thoracic Surgery the First Affiliated Hospital of Soochow University Suzhou China; ^3^ Department of Cardiothoracic Surgery the Affiliated Suzhou Science & Technology Town Hospital of Nanjing Medical University China

**Keywords:** anti‐PD‐1 immunotherapy, CNOT4, cytolytic T lymphocytes, lymphokines, NSCLC

## Abstract

The use of immune checkpoint inhibitors that target programmed cell death‐1 (PD‐1) has been proposed for the treatment of advanced non‐small cell lung cancer (NSCLC). However, in clinical trials, cumulative response rates to anti‐PD‐1 treatment were approximately 20% in patients with NSCLC. CCR4‐NOT transcription complex, subunit 4 (CNOT4) is a RING finger protein with E3 ubiquitin ligase activity. We previously reported that CNOT4 may act as a tumor suppressor in NSCLC. Here, we examined whether CNOT4 can enhance the efficacy of anti‐PD‐1 immunotherapy in a model of NSCLC. The association of CNOT4 and overall survival was analyzed using datasets from The Cancer Genome Atlas (TCGA). Tumor models were established by subcutaneously implanting tumor cells line (A549 cell) into mice. CNOT4 was observed to be positively associated with relapse‐free survival and overall survival in patients with NSCLC. CNOT4 overexpression suppressed tumor growth *in vivo* and enhanced the effect of anti‐PD‐1 immunotherapy, which was accompanied by increased CD3^+^ and CD8^+^ T lymphocyte infiltration and higher interferon‐γ and tumor necrosis factor‐α levels. In conclusion, CNOT4 may enhance the efficacy of anti‐PD‐1 immunotherapy and may have potential as a prognostic marker for NSCLC, or as a combinational target with anti‐PD‐1 treatment for patients with NSCLC.

## Graphical abstract

CNOT4 was observed to be positively associated with relapse‐free survival and overall survival in patients with NSCLC. CNOT4 may enhance the efficacy of anti‐PD‐1 immunotherapy and may have potential as a prognostic marker for NSCLC, or as a combinational target with anti‐PD‐1 treatment for patients with NSCLC.

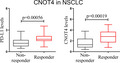

**Abbreviations**
CNOT4: CCR4‐NOT transcription complex, subunit 4
CNOT4‐OE: CNOT4‐overexpressing
CTL: cytotoxic T lymphocytes
CTLA‐4: cytotoxic T lymphocyte antigen 4
E3: E3 ligases
GSK3β: glycogen synthase kinase 3β
NSCLC: non‐small cell lung cancer
OS: overall survival
PD‐1: programmed cell death‐1 √

Immune surveillance is crucial for hosts to protect against tumor progression via secreting cytokines and modulating immune effector cells [1, 2]. However, during tumor progression, tumor variation often results in decreased immunogenicity, increased resistance to immune effector cells, and eventually immune escape [1, 3]. Immunotherapy demonstrated that the immune system could be employed to resist human cancers through targeting immunosuppressive cytokines, regulatory T cells (Tregs), immune checkpoints to generate efficient antitumor immunity [4, 5]. In advanced non‐small cell lung cancer (NSCLC), it is promising to use immune checkpoint inhibitor that targets programmed cell death‐1 (PD‐1) [4, 6, 7]. However, in clinical trials, cumulative response rates to anti‐PD‐1 treatment were approximately 20% in patients with NSCLC [8, 9, 10]. Therefore, it is one of the critical challenges to discover the underlying molecular mechanism that regulates the response to anti‐PD‐1 therapy in NSCLC.

CCR4‐NOT transcription complex, subunit 4 (CNOT4) is a RING finger protein with E3 ubiquitin ligase activity. Although CNOT4 has been demonstrated to associate with viral RNA replication [11] and transcription regulation [12], it remains largely unknown whether CNOT4 was involved in cancer development. Our previous data showed CNOT4 was downregulated in lung cancer tissues as comparing with normal tissues. CNOT4 overexpression significantly attenuated cell growth and metastasis‐associated characteristics *in vitro*. Besides, CNOT4 increased cytotoxicity of cytotoxic T lymphocytes (CTLs) to lung cancer cells, suggesting a potential effect of CNOT4 on regulating antitumor immune responses. To date, no studies have demonstrated the effect of CNOT4 in the clinicopathological significance of anti‐PD‐1 immunotherapies.

In this study, we first identified the correlation between the expression of CNOT4 and recurrence‐free survival and overall survival. Further, we presented the relationship of CNOT4 expression and the efficacy of anti‐PD‐L1 immunotherapy. Finally, we identified the role of CNOT4 in enhancing anti‐PD‐1/PDL‐1 immunotherapy in tumor‐bearing model. Our findings demonstrated the significant role of CNOT4 in tumor growth *in vivo* and the effects of CNOT4 in modulating the sensitivity of anti‐PD‐1 treatment.

## Methods

### Clinical samples

Tumor tissues were collected from metastatic NSCLC patients who received anti‐PD‐L1 treatment. Subsequently, these tissues were fixed and sectioned for subsequent immunohistochemistry staining. This study was conducted at the First Affiliated Hospital of Soochow University and was approved by the ethics committee of First Affiliated Hospital of Soochow University. All procedures performed in studies involving human participants were in accordance with the ethical standards of the institutional and/or national research committee and with the 1964 Helsinki declaration and its later amendments or comparable ethical standards. All participants in this study were informed and given a written consent.

### Cell culture

Control or CNOT4‐overexpressing (CNOT4‐OE) A549 cells were constructed and were maintained in DMEM (HyClone, Logan, UT, USA) medium accompanying with 10% fetal bovine serum (FBS; Gibco, Grand Island, NY, USA), penicillin, and streptomycin (Gibco).

### Mice

C57BL/6 mice were purchased from the SLAC (Shanghai, China) and were housed in a SPF grade animal facility. Four‐week‐old mice were injected subcutaneously with control or CNOT4‐overexpressing (CNOT4‐OE) A549 cells. On day 10 after tumor implantation, tumors became visible. Anti‐mouse PD‐1 antibody (Bristol‐Myers Squibb, New York, NY, USA) was then used as a treatment regimen at the dosage of 100 μg/mice on day 10, 12, 14, 16 [13]. Meanwhile, the relative IgG isotype control was used as control. Tumor volumes were detected every 3 days. Tumor weight was examined on day 27 after excision. Tumor volume was calculated: 1/2 × length × width^2^. The study was approved by the ethics commitment of First Affiliated Hospital of Soochow University.

### Immunohistochemistry

Immunohistochemistry was performed following a standard protocol. Briefly, 5 µm‐thick sections were deparaffinized and rehydrated. Then, the antigen retrieval was performed in Diva Decloaker RTU (Biocare Medical, Concord, CA, USA) for 10 min. Following antigen retrieval, slides were treated using a peroxidase blocking solution (Dako, Agilent, Santa Clara, CA, USA). Next, primary antibodies were incubated overnight at 4 °C. After primary incubation, the corresponding horseradish peroxidase‐conjugated secondary antibodies were used and developed by 3,3′‐diaminobenzidine (DAB; Dako). The slides were captured by a microscope under a magnification of 400×. The primary antibodies included anti‐KI67, anti‐PD‐L1, and anti‐CNOT4.

### Flow cytometry analysis

Single‐cell suspensions were prepared from tumor tissues. In brief, the tumor tissues were digested by Hank's buffer containing 200 U·mL^−1^ DNase type IV, 1 mg·mL^−1^ collagenase, and 0.1 mg·mL^−1^ hyaluronidase at 37 °C for 60 min. Single‐cell suspensions were obtained by passing through 40 μm filter (Falcon). Red blood cells (RBC) were removed using RBC lysis buffer (Roche, Penzberg, Upper Bavaria, Germany). Single‐cell suspensions were stained with antibodies accordingly. Antibodies included PerCP‐Cy5.5‐CD45 (BioLegend, San Diego, CA, USA), FITC‐CD3 (eBioscience, San Diego, CA, USA), and PE‐CD8 (eBioscience).

### RNA isolation and RT‐qPCR

Total RNA was isolated from the tumors excised from mice using an RNAeasy kit (Qiagen, Valencia, CA, USA) according to the manufacture's protocol. One microgram RNA was then used for reverse transcription using a cDNA transcription kit (BioRad, Hercules, CA, USA). Then, Master Mix (Life Technologies, Pleasanton, CA, USA) was used for RT‐qPCR. The Ct data were processed using the average 2^ΔΔCT^ method. *GAPDH* was used as control. *CNOT4* prime: (F: CTCTACAGACTGGCAAGCAGC; R: CCTTTGGCGGTTGTAGTGTG). *GAPDH* prime: (F: TCCCATCACCATCTTCCAG; R: GGTTCACACCCATGACGAAC).

### Data analysis

Kaplan–Meier's analysis of the correlation between CNOT4 expression and overall survival, or relapse‐free survival was acquired from online tool (http://kmplot.com/analysis/), which was based on datasets from The Cancer Genome Atlas (TCGA). Statistical significance was detected by Student's *t*‐test between two groups, and by one‐ or two‐way ANOVA analysis between multiple groups. The *P*‐value < 0.05 was considered significant.

## Results

### Reduced CNOT4 expression predicts poor prognosis in lung cancer

**Fig. 1:**
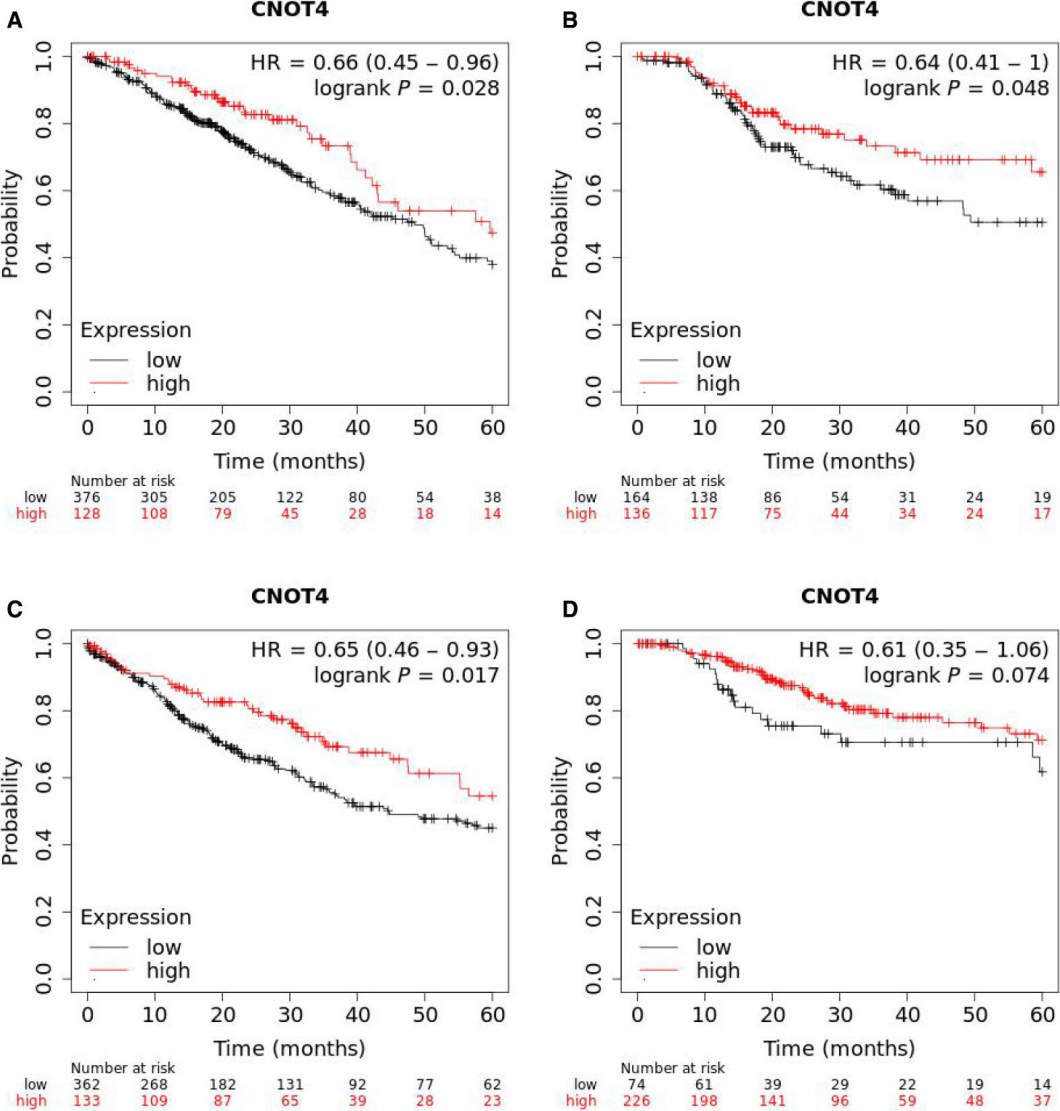
Decreased CNOT4 expression predicts poor prognosis in lung cancer patients. (A) Kaplan–Meier's analysis of the correlation between CNOT4 expression and OS of lung adenocarcinoma patients from TCGA. (B) Kaplan–Meier's analysis of the correlation between CNOT4 expression and RFS of lung adenocarcinoma patients from TCGA. (C) Kaplan–Meier's analysis of the correlation between CNOT4 expression and OS of lung squamous cell carcinoma patients from TCGA. (D) Kaplan–Meier's analysis of the correlation between CNOT4 expression and RFS of lung squamous cell carcinoma patients from TCGA.

To evaluate the correlation between CNOT4 and lung cancer clinically, we assessed the correlation between CNOT4 and overall survival (OS), as well as relapse‐free survival (RFS) using The Cancer Genome Atlas (TCGA) database. As shown in Fig. 1A,B, Kaplan–Meier survival analysis demonstrated that CNOT4 is positively associated with both OS and RFS in lung adenocarcinoma patients. To further confirm this notion, a separate cohort of lung squamous cell patients was used to assess the relationship between CNOT4 and survival. As shown in Fig. 1C,D, patients with high CNOT4 expression were accompanied with the significantly higher OS but not RFS in lung squamous cell carcinoma. Overall, these findings indicated that CNOT4 could be a predictor for OS and RFS in lung cancer.

### The expression of PD‐L1 and CNOT4 is positively correlated with response to checkpoint blockade

**Fig. 2:**
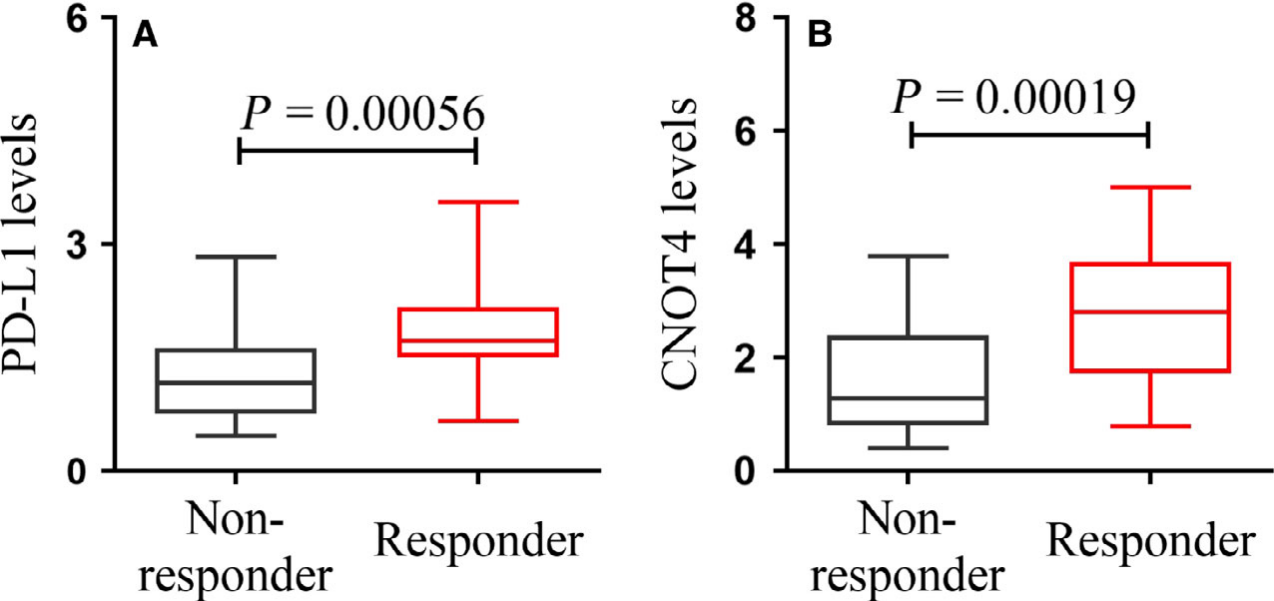
Analysis of the PD‐L1 and CNOT4 signatures and response to checkpoint blockade. Comparison of (A) PD‐L1 and (B) CNOT4 signature between responders and nonresponders. Student's *t*‐test.

To elucidate the contribution of CNOT4 to immune checkpoint blockade therapy in lung cancer, we collected the lung tumor tissues from patients who received anti‐PD‐L1 treatment. From immunohistochemistry staining, we found that PD‐L1, the predictor for response to anti‐PD‐L1/PD‐1 immunotherapy, was higher in patients who responded to anti‐PD‐L1 treatment when comparing with those without response (Fig. 2A). Likewise, the expression of CNOT4 was greater in responding patients than nonresponding ones (Fig. 2B). All these findings demonstrated that CNOT4 may be closely related to anti‐PD‐L1/PD‐1 immunotherapy.

### CNOT4 overexpression enhanced the effect of anti‐PD‐1 immunotherapy in lung cancer

**Fig. 3:**
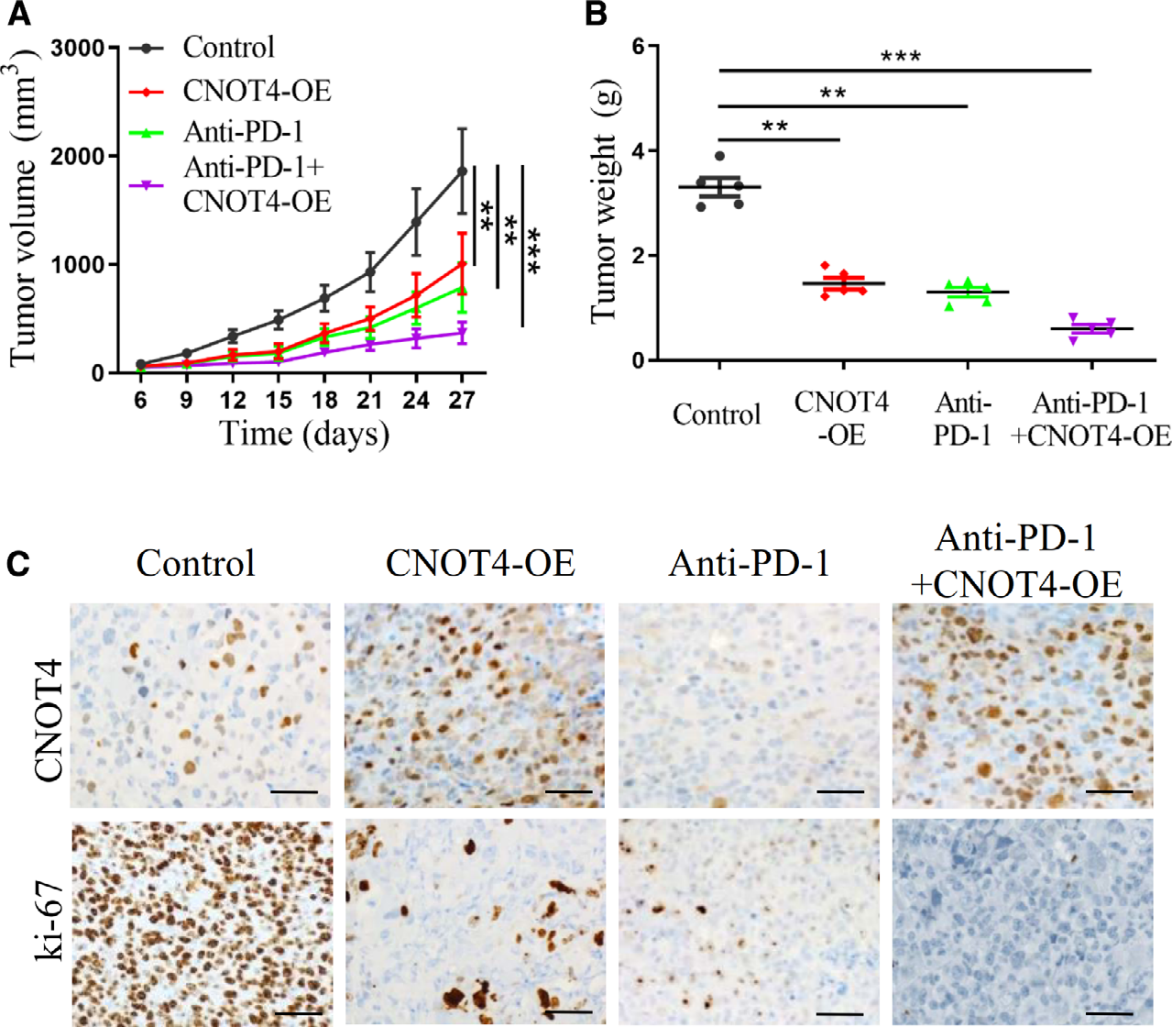
Antitumor effect of CNOT4 overexpression and anti‐PD‐1 antibody alone or in combination. (A) C57BL/6 mice were implanted with 5 × 10^5^ control or CNOT4‐overexpression (CNOT4‐OE) A549 cells and received PD‐1 mAb (100 µg/mice on day 10, 12, 14, 16) treatment or IgG isotype control. Tumor volumes were measured every 3 days. Five mice per group. (B) At day 27, before tumors were excised, tumor weight was measured. (C) Quantifications of CNOT4 and Ki67 count of A549 cell. Scale bar, 50 μm. *P < 0.05, **P < 0.01, ***P < 0.001, Student's *t*‐test.

To further demonstrate the role of CNOT4 in anti‐PD‐1 mediated immunotherapy in lung cancers, we used a tumor‐bearing model, in which control or CNOT4‐overexpression (CNOT4‐OE) A549 cells were subcutaneously implanted into C57BL/6 mice. The tumor volumes were detected every three days. At day 10, the mice bearing either A549 control cells or CNOT4‐OE cells were respectively divided into two groups randomly, in which the mice were received either anti‐PD‐1 mAb (100 µg/mice) or corresponding IgG isotype control treatment. Followingly, at days 12, 14, 16, the mice repeatedly received either anti‐PD‐1 mAb or IgG isotype treatment at the same dose. As shown in Fig. 3A,B, CNOT4 overexpression decreased the tumor volume and weight, which was consistent with our previous *in vitro* data. Similarly, anti‐PD‐1 treatment suppressed tumor growth *in vivo* when compared with the mice receiving IgG isotype control treatment. Notably, combination of CNOT4 overexpression and anti‐PD‐1 treatment dramatically inhibited tumor growth, suggesting that CNOT4 overexpression enhanced the effect of anti‐PD‐1 immunotherapy in lung cancer. Next, the tumors were taken out and were subjected to immunostaining of Ki67, a marker for cell proliferation, and immunostaining of CNOT4 to verify the efficacy of CNOT4 overexpression. As shown in Fig. 3C, CNOT4 overexpression greatly induced CNOT4 expression and decreased Ki67‐positive cells. Meanwhile, the anti‐PD‐1 treatment alone attenuated the number of Ki67‐positive cells and the expression of CNOT4, while CNOT4 overexpression plus the anti‐PD‐1 treatment significantly suppressed cell proliferation. Taken together, we found that CNOT4 overexpression inhibited tumor growth *in vivo*, and CNOT4 improved the efficiency of anti‐PD‐1 treatment in lung cancer.

### CNOT4 overexpression augmented the effects of anti‐PD‐1 via increasing cytotoxic T lymphocyte infiltration

**Fig. 4:**
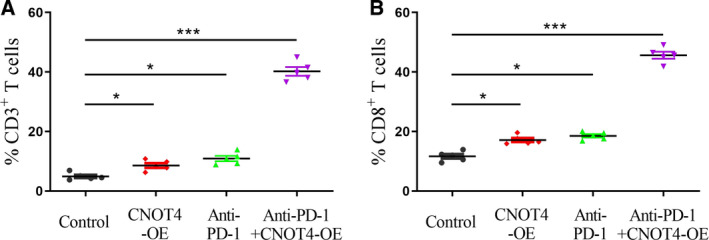
Effect of combinatorial therapy with CNOT4 overexpression and anti‐PD‐1 antibody on tumor. A549 tumor‐bearing mice were treated with single agents or combination of CNOT4‐OE plus anti‐PD‐1 antibody. At the end of the experiment, five tumors were collected from each treatment group, and single‐cell suspensions were prepared and then stained with specific antibodies against immune cell surface markers. Average percentage for positive surface marker was calculated by flow cytometry for each group. (A) FACS analysis of CD3+ total T cells. (B) FACS analysis of CD8+ cytotoxic T cells. Data were presented as mean (±SD) of three independent experiments, *P < 0.05, ***P < 0.001, Student's *t*‐test.

To further decipher the mechanism of CNOT4 on antitumor immunity, we next examined the infiltration of cytotoxic T lymphocytes into tumors by flow cytometry. Fig. 4A showed that the fraction of CD3^+^ cells was higher in CNOT4‐OE tumors than control tumors, and also anti‐PD‐1 treatment in control tumors increased the proportion of CD3^+^ cells, while there was a significant increase of CD3^+^ cells in CNOT4‐OE tumors with anti‐PD‐1 treatment when compared with either CNOT4‐OE alone or anti‐PD‐1 treatment alone. Similar tendency was observed in the fraction of CD8^+^ cells (Fig. 4B). In conclusion, CNOT4 overexpression increases the infiltration of cytotoxic T lymphocytes and raises the efficiency of anti‐PD‐1 treatment.

### CNOT4 overexpression increased expression of lymphokines

**Fig. 5:**
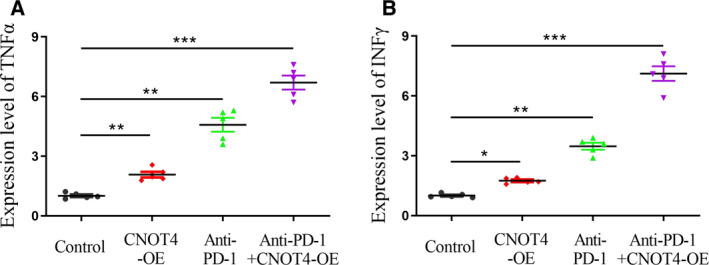
Effect of combinatorial therapy with CNOT4 overexpression and anti‐PD‐1 antibody on cytokine expression. RNAs isolated from A549 tumors from different treated mice were subjected to RT‐qPCR analysis. Expression profiles of cytokines are shown for each treatment group. (A) TNFα. (B) INFγ. Data were presented as mean (±SD) of three independent experiments, *P < 0.05, **P < 0.01, ***P < 0.001, Student's *t*‐test.

Given that lymphokines secreted by cytotoxic T lymphocytes were an effective way to mediate their cytotoxicity, we next detected the expression of interferon (IFN)‐γ and tumor necrosis factor (TNF)‐α by qPCR analysis. Figure 5A showed that TNF‐α expression was increased after CNOT4 overexpression, or after anti‐PD‐1 treatment, while the combination of CNOT4 overexpression and anti‐PD‐1 tremendously enhanced the level of TNF‐α expression. Similarly, the IFN‐γ was upregulated after CNOT4 overexpression, or anti‐PD‐1 treatment, and was highest expressed in the combination group of CNOT4 overexpression and anti‐PD‐1 treatment (Fig. 5B). Collectively, these findings demonstrated that CNOT4 overexpression could increase the expression of lymphokines, including TNF‐α and IFN‐γ, and its combination with anti‐PD‐1 treatment would immensely augment the expression of lymphokines.

## Discussion

Immune checkpoint inhibitors are currently approved to treat multiple types of cancers, which represents a crucial advance in cancer treatment [14]. Cytotoxic T lymphocyte antigen 4 (CTLA‐4) and PD‐1/PD‐L1 are two major pathways that were targeted by immune checkpoint inhibitors, such as monoclonal antibodies or associated molecules [15]. In NSCLC, PD‐1 blockade therapy has been demonstrated impressive durable therapeutically responses [16]. However, only a small fraction of patients had a lasting response. Therefore, it is urgent to understand the underlying molecular mechanism. In this study, we identified a novel determinant, CNOT4 (an E3 ubiquitin ligase) in regulating therapeutic sensitivity of the anti‐PD‐1/PD‐L1 axis.

There are many factors involved in regulating the sensitivity to PD‐1 blockade. PD‐L1 expression level could be a biomarker for response to anti‐PD‐1 treatment [17, 18], and the mutational landscapes have also been characterized to predict the sensitivity to anti‐PD‐1 treatment in NSCLC. Accumulated somatic nonsynonymous mutation burden was associated with improved response to pembrolizumab (anti‐PD‐1) treatment [19, 20]. Accordingly, smoking or DNA repair induced specific mutational signatures could also be a biomarker for response to PD‐1 pathway blockade as well [20]. Besides, mutations in some oncogenes and tumor suppressor genes may have the ability to modulate the immune microenvironment. For instance, enhanced sensitivity to anti‐PD‐1 treatment was observed in lung adenocarcinoma patients who carried TP53 and/or KRAS mutations [21]. In EGFR‐driven lung adenocarcinomas, EGFR pathway activation limited antitumor immunity by upregulating expression of PD‐1, PD‐L1, and inflammatory cytokines, as well as down‐regulating CTLs, which drives immune escape [22]. Moreover, Biton *et al*. [23] demonstrated that combinational use of EGFR, TP53, and STK11 mutations, and PD‐L1 expression represented productive biomarkers to predict responders to anti‐PD‐1 therapy. Therefore, it is a great challenge to find out the predictive markers for the effective response to anti‐PD‐1 treatment. Here, we identified CNOT4 expression is positively correlated with the overall survival of lung cancer patients, suggesting an inhibitory effect of CNOT4 in lung cancer. More importantly, we found that CNOT4 was highly expressed in responders to anti‐PD‐1 treatment, which was consistent with PD‐L1 expression level. However, more evidence is needed to demonstrate whether CNOT4 is a predictor for therapeutic response to anti‐PD‐1 antibodies in an independent cohort.

As previously discussed, high PD‐L1 expression levels in tumor tissues might positively correlate with the sensitivity to anti‐PD‐1 immunotherapy. Although the underlying mechanism of this notion remains unknown, it suggested that the processes controlling protein enrichment and activity may be involved in modulating sensitivity to anti‐PD‐1 immunotherapy. Indeed, ubiquitination mediated degradation, an important process that regulates protein abundance and function, has been shown to regulate antitumor immune responses. In particular, E3 ligases (E3) control the specificity of recognition in the ubiquitination complex and have been found involved in T‐cell tolerance, antitumor immunity, and autoimmunity as well. For example, β‐transducin repeat‐containing protein/cullin 1 was involved in glycogen synthase kinase 3β (GSK3β) induced PD‐L1 decrease by mediating phosphorylation‐dependent proteasome degradation [24]. Besides, cullin 3‐based E3 ligase participated in the process that cyclin D–CDK4 decreased the abundance of PD‐L1 via proteasome‐mediated degradation [25]. These findings indicated that E3 ubiquitin ligases play an important role in reducing PD‐L1 abundance and then controlling cancer immune surveillance. In this study, our results demonstrated that both CNOT4 and PD‐L1 were upregulated in patients who responded to anti‐PD‐1 treatment. The direct correlation of CNOT4 and PD‐L1 expression remains to be determined in the future. Meanwhile, whether CNOT4 regulates PD‐L1 expression stays elusive.

To further explore the role of CNOT4 in restraining tumor growth and regulating anti‐PD‐1/PD‐L1 blockage, the xenograft model was developed. As expected, CNOT4 overexpression inhibited tumor growth and proliferation *in vivo*, which is consistent with our previous *in vitro* data. Surprisingly, CNOT4 overexpression enhanced the effect of anti‐PD‐L1 therapy. PD‐1/PD‐L1 axis is suggested to maintain self‐tolerance via suppressing activation and proliferation of T cells [26]. We next found that CD3^+^ and CD8^+^ T lymphocytes were increased in CNOT4‐overexpressed tumors and anti‐PD‐L1 treated control tumors when compared with control tumors. The combinational use of CNOT4 overexpression and anti‐PD‐L1 blockage markedly enhanced the fractions of CD3^+^ and CD8^+^ T lymphocytes. Lymphokines TNF‐α and IFN‐γ were proven to be correlated with the cytotoxicity of CD8^+^ tumor‐infiltrating lymphocytes [27]. We next detected lymphokine expression. Likewise, TNF‐α and IFN‐γ were also profoundly upregulated in combinational use of CNOT4 overexpression and anti‐PD‐L1 treatment. These data showed that CNOT4 overexpression inhibits T‐cell tolerance and subsequently raises the sensitivity to anti‐PD‐1 treatment. However, how CNOT4 regulated the activity of T‐cell response to tumor cells is still unclear.

## Conclusion

Here, we showed that CNOT4 was positively related to relapse‐free survival and overall survival in lung cancer. CNOT4 was closely associated with anti‐PD‐1 immunotherapy and could serve as a possible prognostic marker for response to immunotherapy targeting the anti‐PD‐L1/PD‐1 axis. In the xenograft model, CNOT4 overexpression restrained tumor growth and enhanced the effect of anti‐PD‐L1 targeted immunotherapy, which was accompanied by more T lymphocyte infiltration and higher lymphokines. In conclusion, our findings suggest that CNOT4 functions as a tumor suppressor and could be a combinational target with anti‐PD‐L1/PD‐1 blockage.

## Conflict of interest

The authors declare no conflict of interest.

## Author contributions

BZ, SH, HM, and SC conducted the experiments and analyzed the data. BZ and SH conceived the study. HM and SC coordinated and supervised the study. BZ, HM, and SC wrote the manuscript. BZ, SH, HM, and SC approved the publication of this paper and are accountable for all aspects of the work and ensure the accuracy or integrity of any part of the work.

## Data Availability

The data could be obtained upon reasonable request the corresponding author.
